# Exploring the mechanism of crashes with automated vehicles using statistical modeling approaches

**DOI:** 10.1371/journal.pone.0214550

**Published:** 2019-03-28

**Authors:** Song Wang, Zhixia Li

**Affiliations:** Center for Transportation Innovation, Department of Civil and Environmental Engineering, University of Louisville, Louisville, Kentucky, United States of America; University of British Columbia, CANADA

## Abstract

Autonomous Vehicles (AV) technology is emerging. Field tests on public roads have been on going in several states in the US as well as in Europe and Asia. During the US public road tests, crashes with AV involved happened, which becomes a concern to the public. Most previous studies on AV safety relied heavily on assessing drivers’ performance and behaviors in a simulation environment and developing automated driving system performance in a closed field environment. However, contributing factors and the mechanism of AV-related crashes have not been comprehensively and quantitatively investigated due to the lack of field AV crash data. By harnessing California’s Report of Traffic Collision Involving an Autonomous Vehicle Database, which includes the AV crash data from 2014 to 2018, this paper investigates by far the most current and complete AV crash database in the US using statistical modeling approaches that involve both ordinal logistic regression and CART classification tree. The quantitative analysis based on ordinal logistic regression and CART models has successfully explored the mechanism of AV-related crash, via both perspectives of crash severity and collision types. Particularly, the CART model reveals and visualize the hierarchical structure of the AV crash mechanism with knowledge of how these traffic, roadway, and environmental contributing factors can lead to crashes of various serveries and collision types. Statistical analysis results indicate that crash severity significantly increases if the AV is responsible for the crash. The highway is identified as the location where severe injuries are likely to happen. AV collision types are affected by whether the vehicle is on automated driving mode, whether the crashes involve pedestrians/cyclists, as well as the roadway environment. The method used in this research provides a proven approach to statistically analyze and understand AV safety issues. And this benefit is potential be even enhanced with an increasing sample size of AV-related crashes records in the future. The comprehensive knowledge obtained ultimately facilitates assessing and improving safety performance of automated vehicles.

## Introduction

Technological advancement has brought Autonomous Vehicles (AVs) into reality, with the fact that relationships between vehicles and drivers are likely to be reversed significantly in the next twenty years [1]. To give AV technology a detailed and precise definition, Society of Automotive Engineers (SAE) defines 6 levels of automated driving systems which address the questions pertaining to what extent of driving tasks each level of the automated driving system can support [2]. Twenty-nine states have enacted legislation to regulate AVs and approved AVs public tests [3–4]. Recently, public tests of AV have already been underway in several states of the US such as California, Nevada, and Michigan, etc. [5–7]. The AV manufacturers that are testing AVs on public roads are either from traditional vehicle manufacturers (i.e., Toyota, Nissan, and General Motor), or technology companies (i.e., Google, Uber, and Baidu). These AV manufacturers have commonly adopted SAE’s six levels of autonomy. Most of the vehicles currently that are tested on public roads are either Level 3 (conditional automation) or Level 4 (high automation) AVs.

Current automated driving systems that are tested on public roads typically involve human factors as safety drivers are expected to take over the driving in case the automated driving system has some technical issues. For example, AVs would have difficulty in detecting the surrounding objects or making decisions accordingly depending upon the roadway characteristics. In these cases, it is essential for human drivers to take over the driving in an appropriate and timely manner to ensure the safe transition from automated to manual driving and prevent potential AV crashes from happening. Therefore, the AV safety issue is a concern to the general public, government agencies, as well as the AV manufacturers.

In fact, there were AV crashes already happened and some of the crashes have resulted in fatalities of AV drivers or pedestrians [8–10]. Both the National Highway Traffic Safety Administration (NHTSA) and the National Transportation Safety Board (NTSB) investigated fatal crashes onsite and published either preliminary or final reports. It can be concluded from these reports that probable causes of these fatal crashes span from human driver’s’ inattention to driving environment complexity [11], ignorance of the take-over request from the vehicle [12], and distraction from some secondary tasks [13]. However, these “probable” causes are ought to be further clarified and finalized in further research.

Although identified as a major reason, what factors cause the AV crash and how large the impact is, still remain unknown. From the perspective of preventing potential AV crashes, what causes AV crashes needs to be investigated comprehensively. With these research questions, a comprehensive investigation of AV crashes’ causes and effects is imperatively needed to understand the mechanism of AV crashes so as to facilitate the prevention of future AV crashes.

In practice, this type of effort is refrained majorly due to the lack of sufficient AV crash data caused by the following reasons:

1. Some of the AV field tests are still underway in the closed course without field test data being published;
2. For the public road tests, most states’ Department of Motor Vehicles (DMV) did not publish the AV crash data or/nor update their crash report formats by adding a specialized section for collecting AV-related information, such as the driving mode of AV when collides (autonomous/ conventional), the faulty party of an AV crash, whether take-over requests have been sent to the human driver, and what kinds of warning cue is employed (visual/ audible/ haptic). This has restrained officers from collecting valuable AV crash data.

Since 2014, the California DMV (CA DMV) has begun to require manufacturers to provide the AV crash report (form OL 316) within 10 business days of the crash [14]. All the reports of AV crashes happened in California were available to the public. As of now, the California DMV has received 113 AV crash reports. The only related study at this moment is that Favarò et al. [15] examined California’s AV crash reports by providing an overview of AV crashes. This research is very meaningful in analyzing the impacts of AV crash and the contributing factors. At the same time, the work is more of a qualitative study, with the mechanism of AV crashes remaining unexplored. It also restrains AV manufacturers from identifying the crash causes also based on crashes involving AVs from other manufacturers, and in turn continuously improving the AV safety by targeting these causes.

In this context, the objective of this research is to comprehensively explore the AV crash mechanism with an attempt to understand its pattern, causes, and impacts from analyzing crash severity and collision type based on the most recent records from the California AV crash database (as of now, published reports are through October 2018).

The relationship among the AV crash severity, collision types, driving mode, roadway characteristics, road users and liability, as well as the relationship among the AV collision types, driving mode, roadway characteristics, road users and liability are to be investigated using a hybrid approach of statistical modeling and classification tree.

## Literature review

### Crash modeling

Typically, the traditional approach for modeling crash has been through collecting crash data for the normal condition or traffic data with converting into traffic conflicts for the pre-crash condition. Then, the data is fed into a modeling method which is suitable for predicting dichotomous outcomes (i.e., crash/no crash; traffic conflict/ no traffic conflict) [16]. However, if the dependent variable, such as levels of crash severity or collision types, has more than two types of outcomes, it is necessary to choose crash modeling methods that allowing the dependent variables to have multiple outcomes.

Among all statistical methods for modeling crash, various forms of logit and probit models have become the primary choices for researchers. Other forms such as the mixed generalized linear model with multiple link functions have been widely used as well. Fountas et al. analyzed the injury severities using a correlated random parameter ordered probit approach with time-variant covariates [17]. Yang et al. conducted a two-step identification of the method of secondary crashes on the freeway by using random effect logit regression model [18]. Guo et al. did a thorough evaluation of the impact of various risk factors on traffic crashes, which are presenting different collision types at freeway diverge areas [19]. A Random Parameters Multivariate Poisson-Lognormal (RP-MVPLN) Model with was developed and compared with an MVPLN model from the perspective of fitting crash data. Also, in another study, Guo et al. investigated the factors that affecting cyclist safety by comparing four types of crash models in terms of goodness of fit [20]. The statistical comparison indicated that Spatial Poisson Lognormal (SPLN) model outperforms the rest of the models.

Besides all statistical approaches, data mining and machine learning techniques have also been employed for analyzing and explaining crash data. Huang et al. examined the interactive effect of mountainous freeway alignment, driving behaviors, vehicle characteristics and environmental factors on crash severity using a classification and regression tree model [21]. Osman et al. proposed a bi-level hierarchical classification methodology to identify different types of secondary tasks that drivers are engaged in using their driving behavior parameters [22]. Sun et al. utilized the Latent Class Cluster (LCC) model as a preliminary tool to identify the major factors that contribute to the crashes [23]. Ding et al. adopted a machine learning approach of Multiple Additive Poisson Regression Trees (MAPRT) to sort the relative importance of attributes in explaining pedestrian crashes [24]. Jeong et al. classified the injury severity in motor-vehicle crashes with high accuracy rate by using multiple classification trees such as decision tree, neural network, gradient boosting model and so forth [25].

### Understanding of automation levels

Generally, an AV is a vehicle that is capable of sensing the driving environment and acting like an agent to drive itself. AVs are combinations of a variety of hardware and software techniques to perceive their surrounding environment, including Light Detection and Ranging (LiDAR) sensor, a radar sensor, cameras, GPS, and computer platforms [26]. AVs are expected to reduce the number of fatal rates caused by human errors. According to the statistical report from NHTSA in 2016, human errors are the major factor contributing to 90% of all fatal crashes [27].

The Society of Automotive Engineers (SAE) defines six levels of driving automation in detail, from Level 0 (No automation) to Level 5 (full automation), which can be used to describe the full range of driving automation features [28]. Existing work has been done, aiming to have a better understanding of the different levels of driving automation. Favarò et al. also indicated the four factors that differentiate each SAE level, which is executing steering and throttles control, monitoring driving environment, and fallback performance [15, 29]. However, the SAE definition adopted by car manufacturers and authorities seems not fully adopted by AV owners. As the crash of a Tesla Model S in 2016 was caused by human driver’s overreliance on Autopilot, despite the fact that Tesla [30] has clearly claimed that “Every driver is responsible for remaining alert and active when using Autopilot and must be prepared to take action at any time”. In this case, some car manufacturers such as Google (now called Waymo), Baidu, and Ford would like to skip Level 3 and focus on “complete the work to fully take the driver out of the loop” [31], which might be a good action to simplify the regulations and make general public to use without concerning safety issues.

### Current practices of AV safety

The existing studies of AV safety were conducted in both field and driving simulator study. Although there are public tests undergoing, many field studies have been restrained in the closed circuit to eliminate the risks of having safety issues. Some of the studies focus on the track of AVs to avoid potential collisions if the trajectory is not correct. Omidvar et al. developed an algorithm in the optimization of trajectories for AV in low demand condition at a closed-course signalized intersection. The algorithm optimizes signal control and provides AVs with optimal trajectories. Field tests confirmed the feasibility of the algorithm, and field deployment for high traffic flow rate condition will be prepared as well [32]. Li et al. developed an integrated local trajectory planning and tracking control framework for AVs with obstacle avoidance. An objective function of considering both safety and comfort performance is formulated for assessing the generated trajectories and selecting the optimal one [33]. Zhu et al. presented a novel speed tracking control approach based on a model predictive control framework for autonomous ground vehicles [34]. Hegedus et al. presented a local trajectory planning method on nonlinear optimization which can generate a dynamically feasible, comfortable, and customizable trajectory for highly automated vehicles [35].

As for the simulation studies regarding AV safety, many researchers deployed driving simulators as the tool to conduct experiments. Their focuses are a degree of trust in automated driving technology, and other human factors such as age. Winter et al. investigated the effects of Adaptive Cruise Control (ACC) and Highly Automated Driving (HAD) on drivers’ workload and situation awareness. They found that the driver of a highly automated car has the possibility to divert attention to secondary tasks [36]. Merat et al. conducted a driving simulator study in comparing the effect of changes in workload on performance in manual and highly automated driving. Findings suggested highly automated driving did not have a deleterious effect on driver performance under the condition of drivers’ attention was not diverted to the distracting secondary tasks. Failing to bring enough sample size of participants is one of the limitations for field AV safety tests. But, the driving simulator study addressed this issue and investigate the topic from a human factor’s perspective. Some interesting findings indicate that older drivers are as good as younger drivers when experiencing automated driving systems [37]. Besides, Körber et al. found older drivers can solve critical traffic events as well as younger drivers [38].

To summarize, for the existing practice regarding AV safety research, both field and driving simulator studies tried to address AV safety issues from perspectives of vehicular control and human factors. However, there is a lack of studies that comprehensively investigate into the mechanism of AV crashes by identifying the contributing factors to all the most recent AV crashes on public roads. In this context, this paper aims at quantitatively investigate into the significant and ruling factors that contribute to AV crashes with various severity levels and collision types.

## Materials and methods

### Data collection

The traditional method for collecting crash data relies on police crash reports, regardless of paper or electronic version. Similarly, the AV crash data that used in this research is collected from the following two major sources:

1. Report of Traffic Collision Involving Autonomous Vehicle (OL 316) [14]: starting 2014, the CA DMV created the specific section for summarizing all the traffic collision reports which involve AVs. This database provides detailed information regarding the collision that occurred when testing automated vehicles on public roads in California (i.e., manufacturer’s information, crash information, serious injuries to people, other associated factors such as weather, lighting, pavement condition). As of October 24, 2018, the CA DMV has received 107 automated vehicle collision reports. Therefore, crashes that occurred only in 2017 and 2018 are used in this research. In total, the CA DMV AV crash database contributed 107 reports of traffic collisions involving AV that are included in the study.
2. Known AV crashes news from nationwide: Except for California, other states do not publish AV crash records officially through their DMV websites. Since the public has been interested in AV testing and safety, news regarding AV crashes was frequently broadcasting via all kinds of media. In this study, some other AV crashes were collected based on both local and national news. In addition, the National Transportation Safety Board (NTSB), which is responsible for investigating the independent accident and advocating safety improvements, published the final report of these crashes involving AVs, which validate the dataset. Therefore, another 6 reports of a traffic crash involving AVs are added to the dataset in this study.

To summarize, a total number of 113 crash records are included in this study. This is by far the most completed AV-related crash data since 2014, with which we can identify and collect from all possible sources. This dataset is further associated with manufacturers information, crash-related information, hardware sensors coverages and other associated factors. This will be further explained in the following section.

### Variables

#### Safety performance measures: Crash severity and collision type

To further understand the mechanism of the crashes involving AVs, crash severity and collision type are selected as the dependent variables in the study. These performance measures are essential components in an accident. There are benefits for autonomous car companies to better understand the mechanism of AV crash in order to improve safety and for governments to form better regulations.

Federal Highway Administrations (FHWA) classified the injury by its scale and definitions [39]. In this study, the KABCO scale would be applied to classify injury levels for all AV crashes. Each injury level is defined as follows: K (Fatality), A (Incapacitating injury), B (Non-incapacitating injury), C (Possible injuries) and O (No injury/ Property damage only).

To better understand the dynamics of the accident, it is necessary to analyze the relative motion of the two vehicles. Milton, Shankar et al. have highlighted the importance of investigation into collision types [40]. Amiri, Nadimi et al. have predicted crash severity on its related collision types using data mining techniques [41].

After examining the crash reports employed in this study, collision types are categorized into “Rear end”, “Sideswipe”, “Angled collision”, and “Run off the road” 4 types.

#### Potential contributing factors

As crash severity and collision type are the dependent variables in the models, the remaining explanatory variables consist of the information retrieved from crash reports. Table 1 summarizes these potential variables that may impact the above components in an AV-involved crash.

**Table 1:** AV crash data variables.

	Variable	Description	Type	Definition	Count (Proportion)
**Performance/** **Dependent Variables**	Crash Severity (CS)	Different levels of crash injuries	Ordinal	K (Fatal)	3 (3%)
				A(Incapacitating injury)	1 (1%)
				B (Non-incapacitating injury)	10 (9%)
				C (Possible injury)	2 (2%)
				O (No injury/Property Damage)	97 (86%)
	Collision Type (CT)	Different types of collision	Categorical	Rear End	69 (61%)
				Sideswipe	25 (22%)
				Angled Collision	10 (9%)
				Run off the road	9 (8%)
**Explanatory Variables / Potential Contributing Factors**	Faulty Party (FT)	The party who is responsible for the crash.	Binary	1 = AV’s fault	15 (13%)
				0 = Not AV’s fault	98 (87%)
	Yielding to pedestrian/cyclist resulted collision or not	Whether the collision was happened due to yielding to pedestrian/ cyclist or not	Binary	1 = Collision was happened due to yielding to pedestrian/ cyclist	12 (11%)
				0 = Collision was happened without due to yielding to pedestrian/ cyclist	101 (89%)
	Roadway Characteristics	Special characteristics and locations identified in AV collision reports	Categorical	Highway	5 (4%)
				Signalized Intersection	70 (62%)
				Stop/Yield signs or behaviors	22 (19%)
				Lane-changing	9 (8%)
				Overtaking	7 (6%)

### Modeling approach

#### Ordinal logistic regression modeling

For identifying the significant factors contributing to crash severity, levels of crash severity can be classified into an order from “K” (fatality) to “O” (least severe injury) based on the injury description reported in the crash reports. The “K” through “O” severity levels follow certain order as “K” being the most severe while “O” being the least se severe. Therefore, we used ordinal (ordered) logistic regression model to analyze the contributing factors to AV severity levels. The model has the following form:

$$P(x=i)=\frac{1}{\left(1+e^{-z_{i}}\right)}$$ (1)

$$z_{i}=a_{i}+\sum_{k}\beta_{k}x_{ik}$$ (2)

Where:

*P*(*x* = *i*) = probability of the AV crash being the i^th^ injury severity level (i follows the order of 1 = K, 2 = A, 3 = B, 4 = C, and 5 = O.)

*z_i_* = a linear function of multiple factors for injury severity level i;

*a_i_* = constant of the linear function when the AV crash has the injury severity level i;

*x_ik_* = *k*^*th*^ variable that can significantly affect the probability of the i^th^ injury level;

*β_k_* = coefficient of the *k*^*th*^ variable.

#### Regression and classification tree (CART) modeling

Using a decision tree to classify a nominal dependent variable is called a classification tree [42].

The classification is a machine learning based approach used for understanding the mechanism of predicting a dependent variable [43]. If the dependent variable is categorical, CART produces a classification tree. If the dependent variable is numerical, CART produces a regression tree. In this study, both crash severity and collision type are considered as categorical variables. CART models are suitable for exploring the following dependent variables:

- Exploring the relationships among the crash severity, collision type, faulty party, whether involving pedestrians/cyclists, and roadway characteristics;
- Exploring the relationships among the collision types, faulty party, whether involving pedestrians/cyclists, and roadway characteristics.

The two basic components of decision tree models are the “root node” and the “leaf node” [44]. The root node is divided into two child nodes with independent variable creating the best homogeneity. The dividing procedure would be repeated until all the data in each node reach their highest homogeneity. The split criterion in the CART method is based on Gini, which is the diversity of a factor. Gini is calculated in the following form:

$$gini=1-\sum_{i}^{n}{p_{i}}^{2}$$ (3)

Where:

*i* = the category of the dependent variable;

*n* = the total number of the dependent variable;

*p* = the percentage of each category in the dependent variable.

Following this sequence, the classification tree can be plotted. The strength of the CART model, compares with other machine learning techniques such as Random Forest, is that “leaf node” that impacting the nominal dependent variables can be quantitatively analyzed.

Normally, the classification models are built from a training dataset in which trends of explanatory and response variables are identified and used to predict the value of the dependent variable for the testing dataset [45]. In this study, these tree graphs can assist car manufacturers to understand the mechanisms of AV disengagement. Furthermore, decision trees models are developed using a package named “rpart” in R studio. The reason why choosing “rpart” to build these decision tree models is due to the function of cross-validation [46], which the training and testing dataset are the same for saving the disadvantage of short sample size.

## Results and discussion

The following analysis is structured in the following way. First, an overview regarding the levels of crash severity and collision types in the different driving mode as well as liability issues is provided. Then, what are the contributing factors and how do they affect the levels of crash severity and collision types are analyzed respectively using the classification tree model and logistic model.

### Analysis of crash severity and collision types distribution

This study first examines the percentage of all collisions reports that automated vehicles were driving with Automated Driving (AD) mode. Among all 113 AV involved crashes, 76 crashes happened with the vehicle driving on AD mode. 37 of these crashes happened with the vehicle driving on conventional mode.

**Fig 1:**
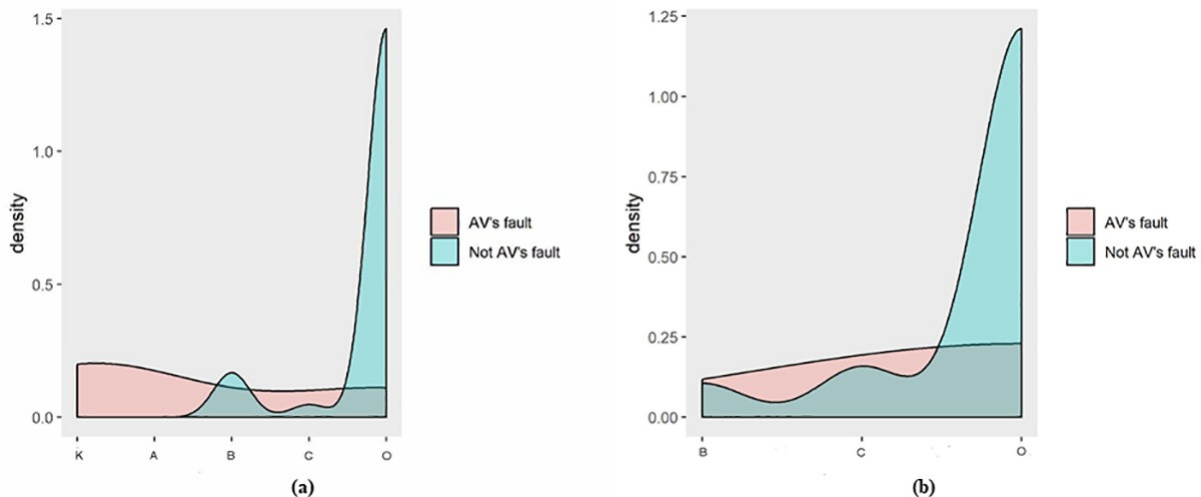
Densities of each level of crash severity in (a) AD mode; (b) Conventional mode.

Fig 1 illustrates the density of each level of crash severity in AD mode or conventional mode in terms of whether AV is responsible for the collision or not. As shown in Fig 1A, if the AV is responsible for the collision, it shows a higher proportion of having a crash severity level of “K” or “C” than the circumstance that AV is not responsible for the collision when driving the automated vehicle with AD mode. This is mainly due to the unexpected behaviors from road users or discarding the take-over request from the AV. Since automated driving is under testing and development, it is necessary to prioritise the safety in order to prevent severe injuries from happening. If the AV is not responsible for the collision, it shows a higher proportion of having a crash severity level of “O”.

As shown in Fig 1B, when drivers manually operate the AV, the probability of having a crash severity level of “K” or “A” decreases compares with AV driving with AD mode.

The ordinal logistic regression is adopted to identify whether AV’s being the faulty party at a crash can significantly impact the level of crash severity. Table 2 summarizes the ordinal logistic regression results for crash severity in AD mode. the variable of faulty party is the significant factor contributing to the crash severity. The result shows that the p-value for “Not AV’s Fault” is smaller than 0.05. It means that liability issue is the significant factor that impacting the injury level in an AV crash at the confidence level of 95%.

**Table 2:** Ordinal logistic model results for crash severity (AD mode).

	B	S.E.	Wald	df	Sig.	95% Confidence Interval		Exp (B)
						Lower Bound	Upper Bound	
Dependent Variables								
Crash Severity (K)	-.341	.793	.185	1	.667	-1.896	1.213	0.711
Crash Severity (A)	.217	.784	.077	1	.782	-1.319	1.752	1.242
Crash Severity (B)	1.881	.925	4.130	1	.042	.067	3.695	6.560
Crash Severity (C)	2.111	.938	5.069	1	.024	.273	3.949	8.256
Independent Variables								
Not AV’s Fault	4.049	.996	16.544	1	.000	2.098	6.001	57.34

The “Not AV’s Fault” has a positive coefficient of 4.049. It suggests that when the AV is operating in AD mode and is not responsible for the crash, the injury level would be significantly lower (57.34 times) than the circumstance when AV is in the AD mode and is responsible for the crash.

The ordinal logistic regression model is also adopted to analyze the crash severity when AV is operating in conventional mode. As summarized in Table 3, the level of crash severity increases significantly if the AV is on conventional mode and is not responsible for the crash.

**Table 3:** Ordinal logistic model results for crash severity (conventional mode).

	B	S.E.	Wald	df	Sig.	95% Confidence Interval		Exp (B)
						Lower Bound	Upper Bound	
Dependent Variables								
Crash Severity (B)	-21.121	.734	828.474	1	.000	-22.559	-19.683	6.560
Crash Severity (C)	-20.082	.493	1.656E3	1	.000	-21.049	-19.115	8.256
Independent Variables								
Not AV’s Fault	-18.556	.000	.	1	.000	-18.556	-18.556	-18.556

**Fig 2:**
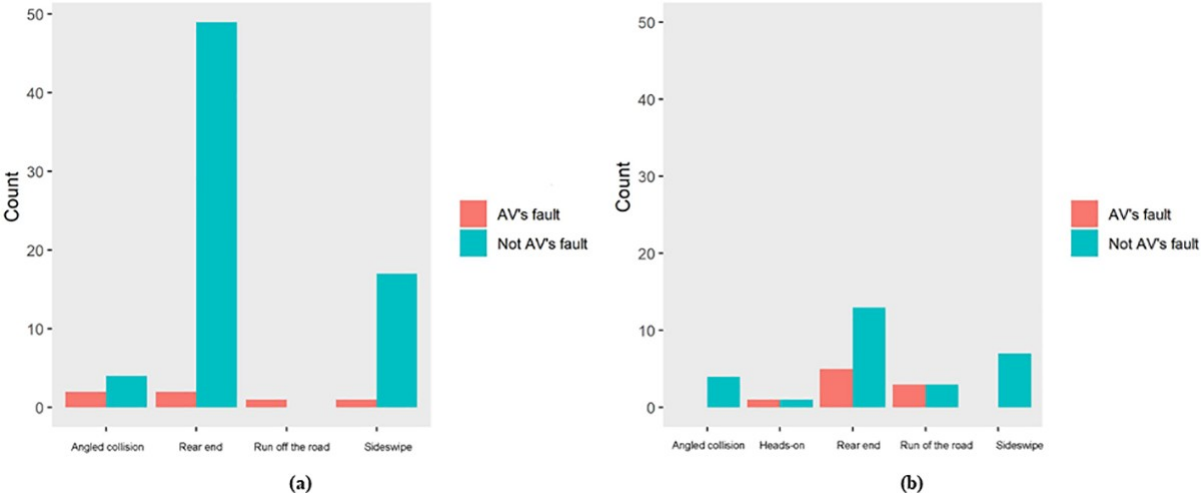
Distribution of each collision types in (a) AD and; (b) Conventional mode.

Fig 2 illustrates the distribution of collision types from the perspectives of both driving modes and liability issues. As shown in Fig 2A, when the crash involves AV and it is on AD mode, it is more likely to be the non-AV’s responsibility rather than AV’s. In more detail, it is found that the AV is rear-ended in most cases compares with other collision types, followed by being sideswiped. For all the cases that AV is driving on conventional mode, as shown in Fig 2B, the AV is found to be rear-ended more times than being sideswiped or collided in angle. Moreover, the AV is found to be responsible for “Rear End” collision more times when it is on conventional mode, compares with when it is on AD mode. This means that when AV is driving itself on the road, it would less likely to rear-end other vehicles compares with the circumstance that AV is driven by human drivers.

### Analysis of AV crash severity

#### Mechanism of various effects on AV crash severity

**Fig 3:**
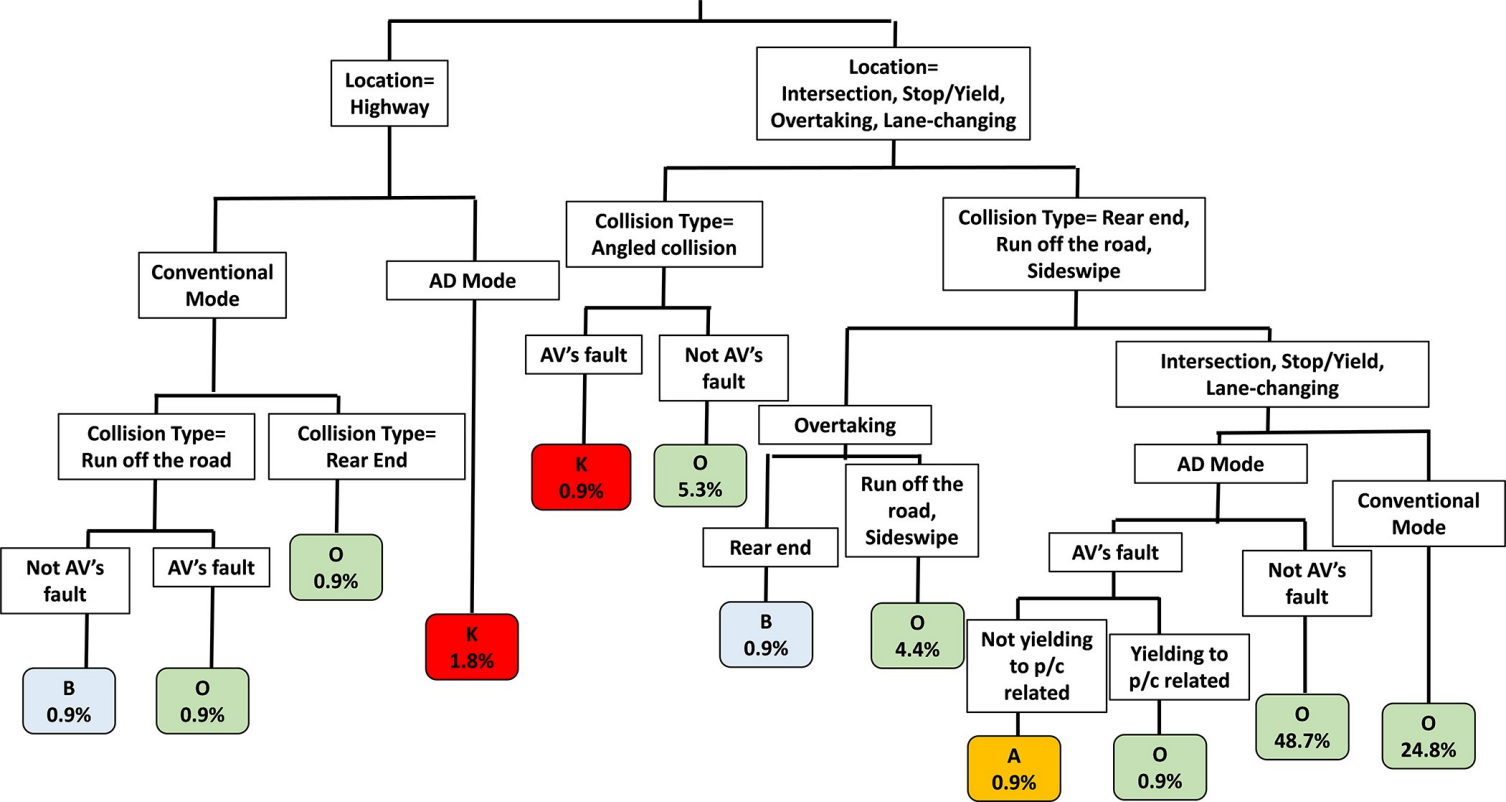
Mechanism of crash severity in collisions with AV involved.

Fig 3 illustrates the relationship between crash severity and potential contributing factors. Variables including driving mode, roadway characteristics, liability, collision type, and whether the crash involving pedestrians/cyclists affect the crash severity. The percentages of observations in classification are also included in Fig 3.

- The findings are summarized as follows: Crashes that took place on the highway are affected by driving modes, collision types, and liability issues. According to the crash database, crashes that happened when the vehicle is on AD mode result in fatalities. Crashes of Tesla in 2016 (Florida) and 2018 (California) are the examples of the fatalities. One common fact from these two fatal crashes is that drivers ignored the warning of taking over from AVs, which means that these two drivers did not take over the driving in an appropriate and timely manner to secure driving safety. This also suggests that AVs are the responsive party for these fatal crashes. In addition, according to the NTSB’s investigation reports of these two fatal crashes, AVs’ speed was 71 mi/h~74 mi/h before making contacts with the object or the semitrailer, which is higher than the posted speed limit of 65 mi/h. Therefore, it is noted that both ignoring the take-over warning and traveling on the freeway with the speed above the speed limit are two major causes to the fatal crashes.
- Crashes that took place on the local roads (i.e., proceeding at intersections, changing lanes, overtaking a vehicle) are affected by driving modes, collision types, liability issues, and whether involves yielding to road users such as pedestrians or cyclists. One fatal AV crash on the local road draws the attention, which is the Uber test AV struck a pedestrian at nighttime in Arizona. According to the NTSB’s investigation, the emergency braking maneuvers are not enabled while the vehicle is on AD mode [13]. Therefore, the AV is responsible for this fatal crash. Despite that not every collision with AV being the responsible party would result in the fatal crash, there is one crash that leads to incapacitating injury, which is the one took place in Utah, 2018. The AV rear-ended a firefighter truck with the AV driver “suffered serious injuries that have deprived her of being able to enjoy life” [47]. A conclusion can be drawn from the above fatal or incapacitating crashes is that when the vehicle is on the AD mode and is the responsive party for the crash, it is likely to have a severe injury.
- To address the issue that AVs brings severer injury than the conventional vehicle, there are many solutions can be found from the perspective of AV technology. Besides, there are also some alternatives can be identified simply according to the current limited AV crash database. As illustrated in Fig 3, as long as AV is not the responsive party for the crash on local roads, the injury level decreases. This is reflected by the fact that 48.7% of the crashes with the vehicle in the AD mode but not being the responsive party has the crash injury of “O”, which these crashes just result in the damage on the vehicle instead of road users. This finding is also consistent with the ordinal logistic regression in the previous section. In addition, switching to manual driving can also be a solution to avoid potential severe crashes brought by automated driving, regardless of driving on the highway or on the local roads. Given the fact that the AV crash database is provided by Level 3 or 4 AVs, it is essential for test drivers to take over driving in a timely manner to avoid severe crashes.

To summarize, it can be concluded that if the vehicle is on AD mode and responsible for the crash, the crash can result in severe injuries (i.e., fatality or incapacitating injuries). The highway is the roadway where most of the severe injuries took place. The current field AV crash data indicates that the injury level decreases when AV is not the responsible party for the crash.

#### Discussion of model accuracy

Table 4 summarizes the classification accuracy of the classification tree illustrated in Fig 3. Overall, the model classifies 91.2% of all the crash data correctly, especially with an accuracy rate of 100% in crash severity of “K”, “A”, and “O”. The prediction accuracies for “B” and “C” are lower. This is due to the small sample size of “B” and “C” crashes. According to Table 1, there are 10 crash records with severity “B” and two crash records with severity “C”, while compared to 97 crashes with severity “O”. The classification method CART tends to have low classification accuracy for one observation (e.g., severity “C” or “B” in the tree) unless the data for this observation has sufficient sample size [48]. This can be the major reason why severity levels “B” and “C” have lower prediction accuracy. It is expected that the prediction accuracy for severity levels “B” and “C” will increase, once more “B” and “C” crashes are added into the analysis in future research.

**Table 4:** Model accuracy of AV crash severity classification tree.

Overall Accuracy: 91.2% (103/113)		Predicted Values				
		K	A	B	C	O
Ground Truth Values	K	100% (3/3)	0	0	0	0
	A	0	100% (1/1)	0	0	0
	B	0	0	20% (2/10)	0	80% (8/10)
	C	0	0	0	0	100% (2/2)
	O	0	0	0	0	100% (97/97)

### Analysis of Collision type of AV crash

#### Mechanism of various effects on AV collision types

**Fig 4:**
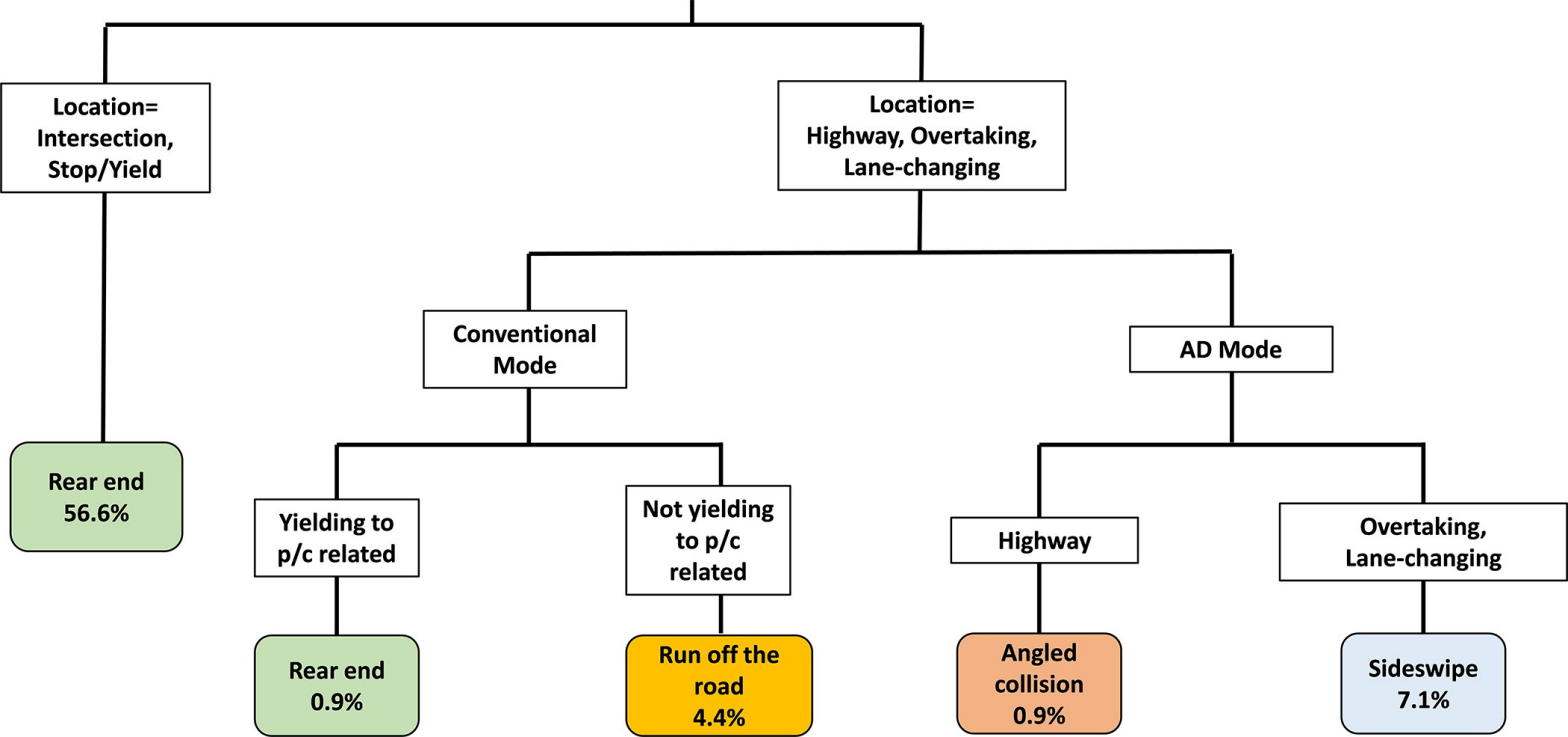
Classification tree of collision type in AD mode.

Fig 4 illustrates the explored classification tree regarding collision types in AV involved crashes. Roadway characteristics, driving modes as well as whether the crash is associated with yielding to pedestrians/cyclists affect the collision types. The percentages of observations in classification are also included in Fig 4.

The findings are summarized as follows:

- The intersection is the place where is most likely to have rear-end collisions than other roadway characteristics, regardless of signalized or unsignalized intersections. This is due to fact that the crash took place when the vehicle is waiting or slowly proceeding at the intersection.
- Collision types on the highway or lane changing are affected by driving modes and whether the crash has pedestrians/cyclists involved. When the vehicle is on AD mode, it has an angled collision occurred on the highway. This finding is reflected by the Tesla crash in Florida, 2016, where the AV struck a vehicle with the right angle and resulted in a fatal crash of the AV driver. Sideswipe is the collision type that when AV is involved with lane-changing.
- When the vehicle is on conventional mode, the collision type is depending upon whether yielding to pedestrians/cyclists or not. If the vehicle is yielding to pedestrians/cyclists, it is more likely to have a rear end collision. This can be explained by the fact that when yielding to these road users, the leading vehicle is going through a process of deceleration. A rear-end collision could happen if the following vehicle fails to provide sufficient deceleration rate accordingly. If the crash has no pedestrians/cyclists involving, the crash is taking place on the highway with the collision type of running off the road.

#### Discussion of model accuracy

Table 5 summarizes the overall and breakdown accuracy of the classification tree model of collision types in these AV involved crashes. Overall, the classification tree of collision types has a total accuracy of 70%. Specifically, 94.2% of all the rear end crashes are classified correctly. The prediction accuracies for “Angled collision” and “Sideswipe” are lower. This is due to the relatively small sample size of “Angled collision” and “Sideswipe”. According to Table 1, there are 10 crash records with “Angled collision” and 25 crashes records with “Sideswipe”. If the database has an insufficient sample size for a certain observation, the CART model tends to have low classification accuracy for this observation [48]. Therefore, this is the major reason why both “Angled collision” and “Sideswipe” have lower prediction rate than other collision types. Once more crashes with collision types being “Angled collision” or “Sideswipe” are collected, the prediction accuracy is expected to increase.

**Table 5:** Model accuracy of collision types in classification tree.

Overall Accuracy: 70.0% (79/113)		Predicted Value			
		Angled collision	Rear end	Run off the road	Sideswipe
Ground Truth Value	Angled collision	10% (1/10)	90% (9/10)	0	0
	Rear end	0	94.2% (65/69)	2.9% (2/69)	2.9% (2/69)
	Run off the road	11.1% (1/9)	33.3% (3/9)	55.6% (5/9)	0
	Sideswipe	0	64% (16/25)	4% (1/25)	32% (8/25)

## Conclusions

The analysis based on statistical and classification tree modeling has successfully identified the contributing factors that impact automated vehicle safety from both perspectives of crash severity and collision types. Particularly, the CART model has revealed the mechanism of automated vehicle-related crashes via visualizing the hierarchical structure of contributing factors to AV crash severity and types.

In conclusions, severe injuries can happen if the vehicle is on automated driving mode and is the major responsible party for the crash. The highway is identified as the location where severe injuries are likely to happen due to high travel speed. Collision types of AV-related crashes are dependent upon the driving mode, location, and whether crashes are associated with yielding to pedestrians/ cyclists. Both ordinal logistic regression and the CART models show consistent results. The resulting hierarchical structure of the AV crash mechanism with knowledge of how the traffic, roadway, and environmental variables can lead to crashes of various serveries and collision types. Although the sample size is limited, the crash database that used in this study contains by far the most complete published crash records with AV involved as of November 2018. The method used in this research provides a proven approach to analyze and understand AV safety issues. And this benefit is potential be even enhanced with an increasing sample size of AV-related crashes records in the future. The comprehensive knowledge obtained in this research can ultimately facilitate assessing and improving the safety performance of current automated vehicles.

With an attempt to ultimately understand the mechanism of AV crash, future research will focus on continuing to collect AV crash data to fit into the CART models used in this manuscript. It is expected to have better prediction accuracy once more AV crashes are added into the analysis in future research. In addition, multiple machine learning based modeling approaches (e.g., Random Forest, AdaBoost, and CHAID) will be employed in modeling the AV crash types and injury severities. The results and prediction accuracy will then be compared with results from the CART model.
